# Editorial: Structural and Dynamic Aspects of Protein Function and Allostery

**DOI:** 10.3389/fmolb.2022.876499

**Published:** 2022-03-10

**Authors:** George P. Lisi, Ivan Rivalta, Vincenzo Venditti

**Affiliations:** ^1^ Department of Molecular Biology, Cell Biology and Biochemistry, Brown University, Providence, RI, United States; ^2^ Dipartimento di Chimica Industriale “Toso Montanari,” Alma Mater Studiorum, Università di Bologna, Bologna, Italy; ^3^ Université de Lyon, École Normale Supérieure de Lyon, Université Lyon 1, CNRS UMR 5182, Laboratoire de Chimie, Lyon, France; ^4^ Department of Chemistry, Iowa State University, Ames, IA, United States; ^5^ Roy J. Carver Department of Biochemistry, Biophysics and Molecular Biology, Iowa State University, Ames, IA, United States

**Keywords:** protein dynamics, spectroscopy, simulations, allostery, enzymes

Chemical signals are routinely propagated through biomacromolecules to modulate the structure of active sites and protein-protein interfaces. However, our understanding of the fundamental mechanisms coupling disparate regions of proteins is limited by the lack of accurate, atomic resolution information on their intrinsic dynamics. Indeed, the pathways most critical to chemical information flow are often intimately linked with the conformational ensembles populated by biomolecules, and spatially distant from traditional catalytic or ligand binding sites. A “holy grail” of biophysical chemistry has been to understand, at the molecular level, how ligand binding information is transmitted through a protein matrix to induce a functional response. Though a majority of information about dynamically-driven biological function comes from lower molecular weight proteins, an exploding number of studies have taken advantage of advances in spectroscopy, electron microscopy, and molecular simulations to synergistically map dynamic pathways that underlie long-range communication in multidomain systems.

The advantage of visualizing the solution ensembles of large biomolecules lies in the potential to leverage flexible hotspots for drug discovery or *de novo* spatial and temporal regulation of protein function. The articles in this Research Topic tackle this knowledge gap by reporting on the structural and dynamic components that govern intra- and inter-domain crosstalk.

Redzic et al. demonstrated an intricate link between micro-millisecond protein dynamics and allostery in biliverdin reductase β (BLVRB). Strikingly, evolutionary differences in amino acid sequence distal to the catalytic site induce a substantial variability in molecular motions that work in concert to regulate BLVRB function. A thermodynamic and kinetic investigation by Dubrow et al. offers insight into the role of protein motions in organizing biomolecular interfaces. Through site-directed mutagenesis, this work captures the per-residue impact on conformational selection during the binding transition state of influenza A nonstructural protein 1 and human p85β. The allosteric interactions of cholesterol with the chemokine receptor CCR3, critical to immune cell trafficking, is characterized by van Aalst and Wylie with circular dichroism and fluorescence polarization. The authors identified cholesterol as a critical mediator of substrate binding and receptor activation that drives signal transduction in GTPase assays. Cole and Igumenova used NMR spectroscopy to explore the interplay between Cd^2+^, Zn^2+^ and the conserved homology 1 (C1) zinc finger domain of protein kinase C, revealing the atomistic structural and thermodynamic properties of the C1 coordination sphere that facilitate competition between divalent metals.

In another study, Purslow et al. highlighted the influence of protein dynamics on the activity of bacterial phosphotransferase Enzyme I by dissecting the contributions of active site flexibility to enzymatic turnover, most notably through the rotameric equilibrium of the catalytic His. Another elegant NMR relaxation study, performed by Zeng et al., revealed an RNA-driven disorder-to-order transition in the N-terminus of a bacterial RNase P, which serves as a dynamic checkpoint to ensure substrate alignment and enzyme activation. Baudin et al. reported an NMR structural study of the SERPINE1 mRNA binding protein (SERBP1), revealing SERBP1 to be intrinsically disordered but capable of sampling several compact conformations. The authors further define its RNA binding preferences and propensity for liquid-liquid phase separation, providing seminal molecular details of its mechanism.

Le et al. carried out a novel structural study of the SpeG N-acetyltransferase, defining the structural basis for an allosteric mechanism that is unique within this enzyme family. This work implicates a dynamic loop, along with several β-strands, as mediators of enzyme activity and lays the foundation for expanded structure-function studies of SpeG. Long-range allostery in α-tryptophan synthase (αTS) is shown by D’Amico et al. to involve networks of flexible residues that propagate ∼25 Å chemical signals. Here, a novel role for surface-exposed residues in modulating dynamic crosstalk in αTS is revealed by NMR and molecular simulations. Skeens et al. and Cui and Lisi explored the intrinsic dynamics of cytokines as a driver for promiscuous and non-overlapping functions. Site-directed mutagenesis, novel structural engineering, and receptor binding demonstrated an intimate link between multi-timescale conformational dynamics and several biological activities.

The computational works contributing to this Research Topic covered various flavors and challenges of modern atomistic simulations, demonstrating the benefits of obtaining information with atomistic resolution that can be directly compared with experimental evidence and highlighting the potential of *in silico* predictions. For instance, Raniolo and Limongelli combined quantum-mechanics and free-energy calculations to improve standard ligand parametrization, allowing enhanced sampling (Funnel-Metadynamics) simulations of the paradigmatic benzamidine/trypsin molecular binding system that elegantly reproduced the high-resolution crystallographic ligand binding mode, providing a very accurate description of the binding mechanism. Hajrediniand and Ghose, instead, showed how enhanced sampling MD simulations could provide insight into the structural mediating role of a conserved “catalytic” residue that inactivates two distantly related kinase families, i.e., bacterial tyrosine and shikimate kinases.

Estarellas et al. computationally assessed changes in the structural and dynamical properties of distinct isoforms of the adenosine monophosphate-activated protein kinase complexes. The comprehensive analysis of molecular dynamics simulations, also involving network theory tools, enabled characterization of key molecular factors that mediated activation of pan-activator PF-739, identifying distinctive features that correlate with the affinities of different isoforms. Massi and Morgan also combined molecular dynamics simulations with network analysis to show that substrate specificity in the enzymatic activity of the oligosaccharyltransferase of *Campylobacter lari* is regulated by modulation of dynamic allosteric pathways. Finally, Pacini et al. provided a perspective on the future challenges of network theory calculations aimed at elucidating the link between the information encoded in protein primary sequences, their dynamics and functions. Jernigan and Kumar proposed, instead, the use of elastic network models to infer the dynamics of a variety of proteins (with known bound and unbound structures) by observing the transfer of fluctuations among distant regions upon binding of an allosteric ligand.

Overall, the collection of manuscripts selected for this special issue of *Frontiers in Molecular Biosciences* highlights once more the power of combining computational and experimental approaches to characterize protein allostery with atomic resolution. We expect that further strengthening and exploiting such synergy will be instrumental toward a complete dissection of the structure/dynamics/function relationship in proteins and the development of predictive tools to identify allosteric networks and hot spots for drug design.

